# BMI-Associated Anti-Apolipoprotein A-1 Positivity in Healthy Adults after mRNA-Vaccination against COVID-19

**DOI:** 10.3390/vaccines11030670

**Published:** 2023-03-16

**Authors:** Roman Máčalík, Marek Petráš, Alexander M. Čelko, Petr Chmátal, Jakub Tlapák, Pavel Dlouhý, Jana Malinová, Ivana Králová Lesná

**Affiliations:** 1 Third Faculty of Medicine, Charles University, 100 00 Prague, Czech Republic; 2 Institute of Aviation Medicine, 160 00 Prague, Czech Republic; 3 Královské Vinohrady University Hospital, 100 34 Prague, Czech Republic; 4 Institute for Clinical and Experimental Medicine, 140 21 Prague, Czech Republic; 5 First Faculty of Medicine, Charles University and Military University Hospital, 121 08 Prague, Czech Republic

**Keywords:** anti-apolipoprotein A-1, autoantibody, mRNA vaccination, BMI, obesity

## Abstract

Elevated anti-apolipoprotein A-1 (AAA1) antibody levels associated with cardiovascular risk have been observed in previously SARS-CoV-2-infected or COVID-19-vaccinated individuals. Since patient safety is generally a priority in vaccination, we sought to investigate AAA1 antibody levels in healthy adults after mRNA vaccination. We conducted a prospective cohort study in healthy adult volunteers recruited from military workers of the Transport Air Base in Prague who had received two doses of mRNA vaccines. Anti-apolipoprotein A-1 antibody levels were determined using ELISA from serum samples obtained at three and four time points after the first and second vaccine doses, respectively, within almost 17 weeks of follow-up. The transient AAA1 positivity rate achieved 24.1% (95% confidence interval CI: 15.4–34.7%), i.e., 20 out of 83 participants had at least one positive post-vaccination sample, with a repeat positivity confirmed in only 5 of them. This rate was associated with a BMI > 26 kg/m^2^, as documented by an adjusted odds ratio of 6.79 (95% CI: 1.53–30.01). In addition, the highest positivity rate of 46.7% (21.3–73.4%) was observed in obese subjects with >30 kg/m^2^. Since the incidence rate of AAA1 positivity remained unchanged after the first and second vaccine doses, any relationship between AAA1 positivity and mRNA vaccination was inconclusive. The present study showed a transient AAA1 positivity rate associated with overweight or obesity without a proven association with mRNA vaccination.

## 1. Introduction

As with any vaccine, a crucial aspect of the new COVID-19 vaccines using mRNA technology is their safety. The mRNA vaccine is able to act on the immune system much more effectively than the current commonly used killed or live vaccines, as demonstrated by high levels of the humoral, cellular or cytokine-mediated immune response [1,2,3].

Non-COVID-19 vaccines are not a trigger of an autoimmune response, as documented by a robust meta-analysis including 144 studies, published in 1968–2019 and designed to investigate any potential association between vaccination and autoimmune diseases [4]. However, it is not clear whether the same applies to new mRNA vaccines against COVID-19 not developed a long time ago [4]. However, it is not clear whether this knowledge can be applicable to new mRNA vaccines against COVID-19 created not a long time ago.

The study of mRNA vaccine-induced autoimmune disorders, with data obtained initially from clinical trials (imbalance in the presence of Bell’s palsy in un- and mRNA-vaccinated subjects), continued with observational studies focused on Guillain–Barré syndrome, thrombosis with thrombocytopenia syndrome and other conditions over the first two years of the global vaccination campaign [5,6,7]. Among the other investigated autoimmune disorders, attention should be given to post-vaccination myocarditis or pericarditis, which is diagnosed more often in younger populations, as shown in several meta-analyses [8,9,10].

Another approach to identifying potential risk factors (including mRNA vaccination) which could increase the occurrence of autoimmune disease, involves the search for certain markers or autoantibodies. A recent study hypothesizing about increased levels of anti-apolipoprotein A-1 antibodies (AAA1) after mRNA vaccination against COVID-19 in rheumatoid arthritis patients as well as in healthy adults was, in fact, based on similar outcomes reported in subjects after SARS-CoV-2 infection [11,12].

Increased AAA1 levels have been mainly shown in autoimmune disorders associated with increased cardiovascular risk, such as systemic lupus erythematosus, primary antiphospholipid syndrome, and in patients with rheumatoid arthritis, acute coronary syndromes and severe carotid stenosis [13,14,15,16,17]. We therefore decided to determine AAA1 levels in our study designed to assess the safety of mRNA vaccination against COVID-19 in healthy adults.

## 2. Materials and Methods

### 2.1. Study Design

This study was designed as a prospective cohort study conducted exclusively in healthy adult volunteers aged 18–55 years, recruited from military workers of the Transport Air Base in Prague. Unvaccinated participants, independently of sex and previous SARS-CoV-2 infection, were scheduled for vaccination against COVID-19 and agreed to be enrolled by signing a written informed consent. The study protocol was approved by the Ethics Committee of the Third Faculty of Medicine, Charles University in Prague (No. 0804/21), Czech Republic.

**Figure 1:**
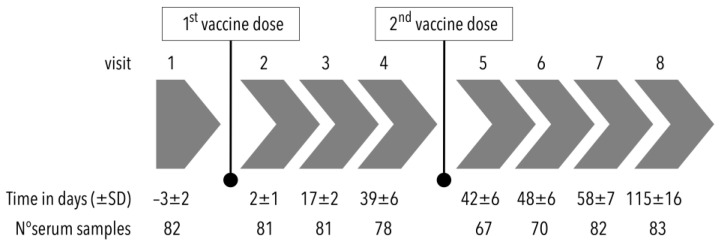
Flowchart of the study design (SD: standard deviation).

Before vaccination, enrolled volunteers underwent a physical examination, including their medical history, and had a total of 8 follow-up visits to have their serum samples drawn and to monitor adverse events including SARS-CoV-2 infection (Figure 1). The serum samples were stored at about −80 °C and assayed in a central laboratory (laboratory of District Hospital at Havlíčkův Brod, Czech Republic) at the end of the study.

### 2.2. Study Endpoints

The primary endpoint of the present study was AAA1 levels determined at each study time point. Secondary endpoints, i.e., antinuclear antibodies (ANA), anti-neutrophil cytoplasmic antibodies (ANCA) and specific antibodies against the spike protein (anti-S IgG), were measured at the beginning of the study, before the second dose administration and at the end of the study.

The levels of AAA1 antibodies were determined using a commercially available Anti-apoA1 antibody ELISA kit (Cusabio Technology, Wuhan, China) according to the manufacturer’s instructions. The AAA1 IgG levels with a manufacturer-defined positivity of ≥2.1 were calculated from the ratio of optical densities of sample and control sera. The present ANA and ANCA antibodies were determined using indirect immunofluorescence tests with Euroimmun ANCA Granulocyte mosaic and ANA cell nuclei (ANA global test), respectively (Euroimmun Medizinische Labordiagnostika AG, Lübeck, Germany). Specific anti-S IgG antibodies quantified in AU/mL were measured using SARS-CoV-2 IgG II Quant (6S60, Abbott Ireland, Diagnostics Division, Sligo, Ireland).

### 2.3. Statistical Analysis

Demographic characteristics and study variables were reported as proportions, the mean including the standard deviation (SD) and the median with the interquartile range (IQR), unless stated otherwise. Participants’ age and BMI were used as binary variables according to the median values of the study population. Categorical variables were assessed using Fisher’s exact or χ^2^ tests. 

Anti-apolipoprotein A-1 IgG levels expressed with the median were evaluated by non-parametric tests, including multiple comparisons. Furthermore, the proportion of subjects having at least one serological result of AAA1 IgG ≥ 2.1 was defined as the AAA1-positivity (AAA1+) rate. They were used for geometric mean concentrations of specific antibodies against the spike protein, and they were analyzed using parametric tests after log-transformation.

The association expressed by the adjusted odds ratio (aOR) between the AAA1+ rate and covariates was established by logistic regression. The incidence of AAA1+ relative to partial and full vaccination was analyzed using the incidence rate ratio (IRR) including the 95% confidence interval (95% CI). Additional analyses of sensitivity were conducted in sets of subjects with all results of serological assays in order to assess an impact of missing values. As the sample size was not determined in a prior study, the power was tested using the one-sample proportion test. In the present study, our definition of superiority to be met, in terms of the AAA1+ rate in the study population, was that the percentage proportion of subjects with AAA1+ had to be higher than 10% (as an alternative hypothesis).

All tests were two-tailed with the level of significance set at 0.05. Statistical tests and analyses were conducted using Prism 9 (GraphPad Software, Inc., San Diego, CA, USA) and STATA version 17 software (StataCorp, College Station, TX, USA).

## 3. Results

The study was conducted between April and December 2021 in a total of 83 healthy adults (69 men and 14 women) aged 24–55 years. The median age and BMI of the study population, including the IRQ, were 40.4 (11.0) years and 26.0 (16.6) kg/m^2^, respectively. At least one comorbidity was identified in 18 subjects, with the most frequent conditions being hypertension in 10, thyroid disorders in 4, and dyslipidemia in 3 volunteers. All subjects were fully immunized with mRNA vaccines, either the BNT162b2 (68 subjects) or the mRNA-1273 vaccine (15 subjects). A total of 28 subjects were previously infected by SARS-CoV-2, with a mean interval ± SD of 146 ± 56 days before immunization. New infection or re-infection was not reported during follow-up.

At the beginning of the study, positive ANA antibodies were detected in 10 subjects, including two of those with a thyroid disorder. This was no longer true in two subjects immediately before the second vaccine dose and in another four participants at the end of the study. Another three and six subjects had positive ANA after the first and second vaccine dose, respectively. Anti-neutrophil cytoplasmic antibodies were not detected in any serum sample both at baseline and after partial and full vaccination.

The overall AAA1+ rate reached 24.1% (95% CI: 15.4–34.7%), i.e., 20 subjects had at least one positive serum sample at 16.4 ± 2.3 weeks of mean ± SD follow-up since the start of immunization. The sample size was sufficient to demonstrate a superiority of >10%, as shown by an 85% power of test. AAA1 IgG levels over the cut-off of seropositivity were found at two and three time points in three and two participants, respectively. The pre-vaccination finding of positive AAA1 levels in three subjects was observed again in only one of their samples after the immunization period. The AAA1+ rate was evaluated independently of the number of doses and the time since vaccination. The assessed predictors included sex, age, BMI, smoking status, comorbidities, previous SARS-CoV-2 infection, and pre-vaccination ANA positivity. The predictor-adjusted OR showed that the AAA1+ rate could be associated with BMI and pre-vaccination ANA positivity (Table 1). 

Positive AAA1 IgG levels were more often seen in subjects with a BMI > 26 kg/m^2^ than in those with a lower BMI, as documented by the aOR of 6.79 (95% CI: 1.53–30.01). An additional analysis confirmed that the AAA1+ rates of 28.9% (95% CI: 15.4–45.9%) in 38 overweighted subjects with a BMI of 25–30 kg/m^2^ (*p* = 0.020) and of 46.7% (95% CI: 21.3–73.4%) in 15 obese subjects with a BMI >30 kg/m^2^ (*p* = 0.002) were significantly higher than the rate of 6.7% (95% CI: 0.8–22.1%) in 30 participants with a normal body weight having a BMI < 25 kg/m^2^.

A total of 10 subjects with ANA antibodies detected prior to vaccination achieved an AAA1+ rate of 40.0% (95% CI: 12.2–73.8%), compared to 21.9% (95% CI: 13.1–33.1%) in those without ANA antibodies. Although both rates were not significantly different (*p* = 0.300), the predictor-adjusted odds ratio of 0.10 (95% CI: 0.01–0.93) suggested a potential association (*p* = 0.043).

**Figure 2:**
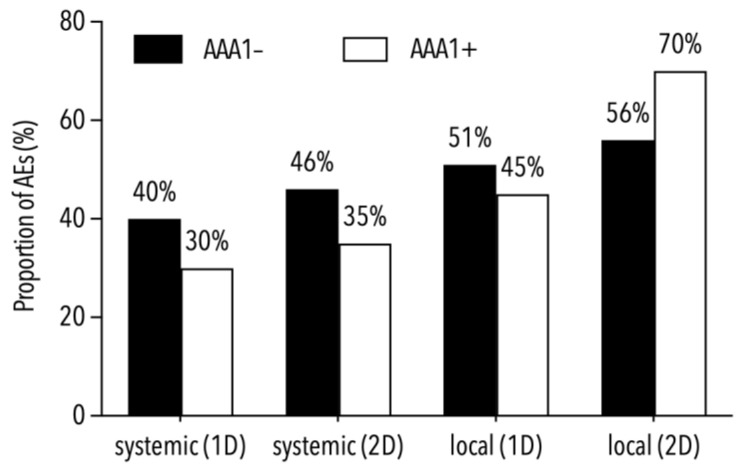
Proportions of local and systemic adverse events (AEs) in subjects with and without AAA1 positivity.

The incidence rate ratio of 0.87 (95% CI: 0.33–2.44) did not indicate different AAA1+ incidence rates in the partially (2.7 cases per 1000 person-days) and fully vaccinated participants (2.3 cases per 1000 person-days) during post-vaccination follow-up. As shown by both AAA1+ rates, i.e., 22% in those vaccinated with BNT162b2 and 33% in those vaccinated with mRNA-1273 (*p* = 0.36), the rate was not dependent on the mRNA vaccine used. AAA1+ subjects reached geometric mean concentrations of IgG antibodies against the spike protein similar to those of the AAA1− ones, i.e., 3900 AU/mL for AAA1+ and 3000 AU/mL for AAA1− subjects before the second dose (*p* = 0.55), and 7900 AU/mL for AAA1+ and 8100 AU/mL for AAA1− subjects at the end of the study (*p* = 0.96). Furthermore, the AAA1+ rate was not linked to either local (pain, swelling or erythema at the injection site) or systemic adverse events (headache, arthralgia, myalgia, chills, fatigue or lymphadenopathy) reported both after the first and second vaccine doses, because their frequencies in subjects with and without AAA1 positivity were not different (Figure 2).

**Figure 3:**
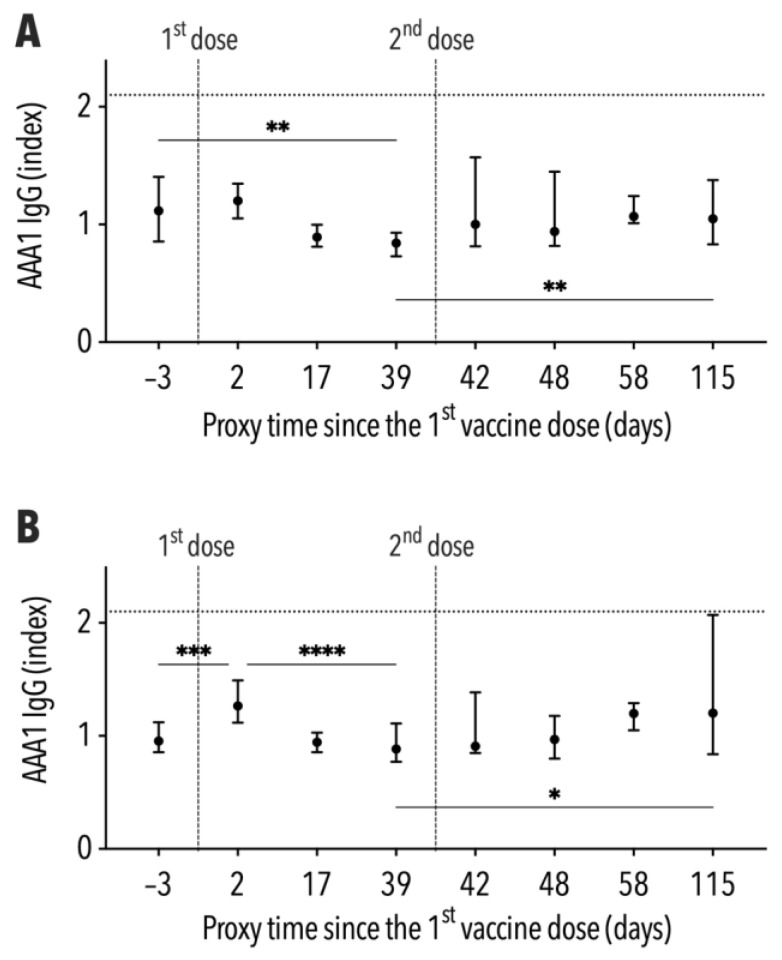
Development of AAA1 levels, including the interquartile range, during follow-up ((**A**)—subjects with BMI ≤ 26 kg/m^2^, (**B**)—subjects with BMI > 26 kg/m^2^). * *p* < 0.05; ** *p* < 0.01; *** *p* < 0.001; **** *p* < 0.0001.

The median of AAA1 IgG levels ranged between 0.85 and 1.21 without exceeding the seropositivity cut-off during follow-up. The antibody levels in participants with a BMI ≤ 26 kg/m^2^ decreased from 1.12 ± 0.55 to 0.84 ± 0.19 between both vaccine doses (Figure 3A). Conversely, the IgG median in subjects with a BMI > 26 kg/m^2^ increased significantly from 0.96 ± 0.26 to 1.27 ± 0.37 within two days of the first vaccine dose, subsequently declining to 0.89 ± 0.34 immediately before the second dose (Figure 3B). The median of AAA1 IgG levels at the end of follow-up was not significantly different from that before vaccination in both BMI-stratified groups. However, a high variability of AAA1 IgG levels was demonstrated by an IQR of 0.84–2.07 in subjects with a BMI > 26 kg/m^2^.

## 4. Discussion

Our study showed an overall AAA1+ rate of 24.1% within 17 weeks of follow-up. This rate was considered transient in most of the subjects, since only 6% of them had positive antibody levels in more than one sample.

When trying to identify factors potentially affecting the transient AAA1+ rate, we documented an impact of overweight and obesity, as shown by rate increases to 29% and 47% in overweight and obese subjects, respectively. Conversely, transient AAA1 positive levels were observed in only 7% subjects with a normal body weight. It seems that transiently elevated AAA1 levels can be encountered more often in overweight or obese subjects after mRNA vaccination. A similar finding of significantly reduced AAA1 levels associated with a post-surgical decrease in BMI was reported in another study [18]. It is well known that obesity can modify the metabolism of high-density lipoproteins, including apolipoprotein A1, and could therefore increase AAA1 IgG levels over the positivity limit, indirectly enhancing cardiovascular disease risk [19,20].

While multivariate analysis suggested a relationship between pre-vaccination ANA levels and the AAA1+ rate after mRNA vaccination, this is still only a possibility, since only a limited number of subjects at baseline, before immunization, had positive ANA, making this issue an interesting topic of future studies.

Whether or not mRNA vaccination can influence the AAA1+ rate was investigated using the incidence rates of positive antibody levels after first- and second-dose administrations. As their ratio did not show different rates after the first and second mRNA vaccine doses, the assumption of an increased risk of AAA1 seropositivity after vaccination was not confirmed. Likewise, no difference in rates was observed in subjects receiving either the BNT162b2 or the mRNA-1273 vaccine, regardless of the number of doses. The levels of antibodies against the S protein elicited by mRNA vaccines, as well as the occurrence of adverse events post-vaccination, were not different between subjects with and without AAA1+. 

We did not document an association between mRNA vaccination and the transient AAA1+ rate, as suggested by a recent study [11]. However, different trajectories of AAA1 levels after vaccination were seen in overweight and obese subjects compared to those with a normal body weight. Several autoantibodies including AAA1 have been implicated in the development and progression of atherosclerosis and its complications. Indeed, AAA1 IgGs have been demonstrated to be a marker and perhaps also a mediator of atherogenesis progression [16,20]. One can only speculate whether or not autoantibodies may, in the long run, indirectly affect the incidence of cardiovascular events after vaccination in overweight and obese persons.

Even with a limited sample size, the power of test of >80% confirmed that a reasonable cohort size demonstrated an anticipated association between the AAA1+ rate and BMI. Admittedly, the underrepresentation of women may be a study limitation. The main limitation of our study results were the missing serum samples, precluding a paired assessment of the course of antibody levels. Nevertheless, our analysis of sensitivity did not change the obesity-associated AAA1+ rate. The absence of standardized assays of AAA1 IgG was replaced by the use of a commercial kit exhibiting a lower sensitivity compared to “homemade” tests [21]. This fact may have contributed to an underestimation of both the AAA1+ rate and antibody levels. However, we are convinced that this possible inaccuracy was not crucial and that similar results can also be plausibly obtained by other serology kits. As our study was conducted with a strictly vaccinated cohort, we are unable to provide the AAA1+ rate for an unvaccinated control group during follow-up. Therefore, an additional limitation of the study is the lack of control groups vaccinated with alternative COVID-19 vaccines or not vaccinated at all. However, we presumed that the effect of vaccination on the positive levels of AAA1 IgG would manifest itself by elevated incidence rates after the second rather than after the first vaccine dose. As this was not the case, an association is unlikely. Therefore, in the future, it could be of interest to study AAA1 levels not only in vaccinated but also in unvaccinated subjects with regard to overweight or obesity. It was our intention to put forward a new hypothesis on the relationship between the AAA1+ rate and BMI, which may, but need not, interact with mRNA vaccination. In our view, studies such as ours could potentially provide an impetus for designing large studies.

## Figures and Tables

**Table 1:** Predictor-specific AAA1 positivity and association with predictors.

Predictors		No Subjects	AAA1+ ^1^ Rate (%)	cOR ^2^ (95% CI ^3^)	aOR ^2^ (95% CI)	P ^4^
Sex	Male	69	27.5	1	1	
	Female	14	7.1	0.20 (0.03–1.66)	0.12 (0.01–1.66)	n.s. ^7^
Age (years)	≤M ^5^	42	21.4	1	1	
	>M	41	26.8	1.34 (0.49–3.69)	2.07 (0.56–7.66)	n.s.
Smoker	Yes	15	13.3	1	1	
	No	68	27.9	2.34 (0.48–11.4)	2.53 (0.46–13.96)	n.s.
BMI (kg/m^2^)	≤M ^5^	42	9.5	1	1	
	>M	41	39.0	6.08 (1.82–20.3)	6.79 (1.53–30.01)	0.012
Concomitant disease	Yes	18	38.9	1	1	
	No	65	20.0	0.39 (0.13–1.21)	0.36 (0.09–1.43)	n.s.
Previous COVID-19	Yes	28	25.0	1	1	
	No	55	23.6	0.93 (0.32–2.67)	0.81 (0.22–2.92)	n.s.
Pre-vaccination ANA+ ^6^	Yes	10	40.0	1	1	
	No	73	21.9	0.42 (0.11–1.68)	0.10 (0.01–0.93)	0.043

^1^ AAA1+: anti-apolipoprotein A-1 positivity; ^2^ cOR/aOR: crude/adjusted odds ratio; ^3^ 95% CI: 95% confidence interval; ^4^ P: *p*-value; ^5^ M: median (age = 42.4 years, BMI = 26 kg/m^2^); ^6^ ANA+: positivity of antinuclear antibodies; ^7^ n.s.: not significant.

## Data Availability

Data available on request due to privacy restrictions. The data presented in this study are available on request from the corresponding author. The data are not publicly available due to a confidential agreement with the Institute of Aviation Medicine, Prague.
